# Exposure to tobacco smoke and childhood rhinitis: a population-based study

**DOI:** 10.1038/srep42836

**Published:** 2017-02-16

**Authors:** Tsung-Chieh Yao, Su-Wei Chang, Wei-Chiao Chang, Ming-Han Tsai, Sui-Ling Liao, Man-Chin Hua, Shen-Hao Lai, Kuo-Wei Yeh, Yu-Lun Tseng, Wan-Chen Lin, Hui-Ju Tsai, Jing-Long Huang

**Affiliations:** 1 Division of Allergy, Asthma, and Rheumatology, Department of Pediatrics, Chang Gung Memorial Hospital and Chang Gung University College of Medicine, Taoyuan, Taiwan; 2 Chang Gung Immunology Consortium, Chang Gung Memorial Hospital and Chang Gung University College of Medicine, Taoyuan, Taiwan; 3 Community Medicine Research Center, Chang Gung Memorial Hospital at Keelung, Keelung, Taiwan; 4 Clinical Informatics and Medical Statistics Research Center, Chang Gung University College of Medicine, Taoyuan, Taiwan; 5 Department of Clinical Pharmacy, College of Pharmacy, Taipei Medical University, Taipei, Taiwan; 6 Department of Pediatrics, Chang Gung Memorial Hospital at Keelung, Keelung, Taiwan; 7 Division of Pediatric Pulmonology, Department of Pediatrics, Chang Gung Memorial Hospital, Taoyuan, Taiwan; 8 Division of Biostatistics and Bioinformatics, Institutes of Population Health Sciences, National Health Research Institutes, Miaoli, Taiwan; 9 Department of Pediatrics, Feinberg School of Medicine, Northwestern University, Chicago, IL, USA; 10 Department of Public Health, China Medical University, Taichung, Taiwan

## Abstract

Exposure to tobacco smoke has been associated with harmful effects on child health. The association between tobacco smoke exposure and childhood rhinitis has not been established in developed or developing countries. We investigated the association between serum cotinine levels and rhinitis in a population sample of 1,315 Asian children. Serum cotinine levels were positively associated with rhinitis ever (adjusted odds ratio [AOR] = 2.95; 95% confidence interval [CI]: 1.15–7.60) and current rhinitis (AOR = 2.71; 95% CI: 1.07–6.89), while the association for physician-diagnosed rhinitis approaching borderline significance (AOR = 2.26; 95% CI: 0.88–5.83). Stratified analyses demonstrated significant association of serum cotinine levels with current rhinitis among children without allergic sensitization (AOR = 6.76; 95% CI: 1.21–37.74), but not among those with allergic sensitization. Serum cotinine levels were positively associated with rhinitis ever (AOR = 3.34; 95% CI: 1.05–10.61) and current rhinitis (AOR = 4.23; 95% CI: 1.28–13.97) among adolescents but not in children aged less than 10 years. This population-based study demonstrates supportive evidence for positive association of tobacco smoke exposure with rhinitis, while the effect is mainly confined to non-allergic rhinitis and more pronounced in adolescents than in young children, highlighting the need for raising public health awareness about the detrimental effects of tobacco smoke exposure on children’s respiratory health.

Allergic diseases commonly develop during childhood as a result of interaction between genetic and environmental factors^1^. For several decades, there has been a remarkable increase in prevalence of childhood allergic diseases such as asthma, allergic rhinitis, and atopic dermatitis (eczema), which has reached epidemic numbers in several countries including Taiwan^2^,^3^,^4^. Specifically, the prevalence of allergic rhinoconjunctivitis in 6–7-year-old children in Taiwan is among the highest around the world^3^, attracting substantial research and public attention. Changes in lifestyle are thought to be at least partly responsible for the allergy epidemics extending beyond the past few decades^2^.

Tobacco use is one of the leading modifiable risk factors for non-communicable diseases^5^. Smoking population has been estimated around 1.3 billion worldwide^6^. It is estimated that approximately one billion people may die due to tobacco-related causes during the twenty-first century^6^. Exposure to environmental tobacco smoke is one of the most common preventable health hazards for children^7^, whose respiratory and immune systems are still growing and defense mechanisms are relatively vulnerable. Using data collected from 192 countries, Oberg *et al*. report that approximately 40% of children, 33% of men, and 35% of women worldwide are regularly exposed to indoor passive smoke^8^. Our recent work estimates that 52.5% of children are exposed to household passive smoke in Taiwan, a country in East Asia^9^. Previous studies assessing the association between tobacco smoke exposure and allergic diseases in children have shown inconclusive results^10^,^11^, which may be explained in part by differences in extent of tobacco smoke exposure, populations, methods, and sample size across studies. Most studies investigating the effects of tobacco smoke exposure on allergic diseases in children have frequently relied on subjective questionnaires, without objective measurement to characterize the extent of tobacco smoke exposure and allergic sensitization. In addition, while the majority of previous studies have been conducted in western countries, little is known about the health impact of tobacco smoke exposure on Asian children.

The present study aimed to assess the relationship of tobacco smoke exposure (measured objectively by serum cotinine levels) with rhinitis in children and adolescents in a well-characterized population-based Asian cohort, the Prediction of Allergies in Taiwanese CHildren (PATCH) study.

## Results

### Subject characteristics

A total of 1,315 children were included in this study. The mean age was 10.3 years (standard deviation [SD]: 2.7) and 49.0% of participants were male. Table 1 presents the demographic and clinical characteristics among the study participants, with 43.7%, 40.8%, and 37.6% of the children having rhinitis ever, current rhinitis, and physician-diagnosed rhinitis, respectively. The mean level of serum cotinine in 1,315 participants was 1.51 ng/mL (SD: 2.99). When stratifying by gender, the mean and corresponding SD of serum cotinine levels were 1.56 (SD: 3.87) ng/ml for boys; and 1.47 (SD: 1.77) ng/ml for girls (*P* = 0.37). The prevalence of rhinitis ever, current rhinitis, and physician-diagnosed rhinitis was 52.3%, 47.3%, and 45.7% for boys; and 35.4%, 34.5%, and 29.8% for girls (all *Ps* < 0.001). In terms of questionnaire response, approximately half of the study participants (54.1%) were currently exposed to passive smoke according to parental questionnaire responses. Serum cotinine levels were significantly higher in subjects with passive smoke exposure than in those without passive smoke exposure (the mean and corresponding SD for subjects with passive smoke exposure: 1.56 ± 2.39 ng/mL; the mean and corresponding SD for subjects without passive smoke exposure: 1.30 ± 0.31 ng/mL; *P* = 0.01). Of note, 0.7% (9/1,315) of study participants were active smokers based on children’s self-report.

### Association between tobacco smoke exposure and childhood rhinitis

**Figure 1:**
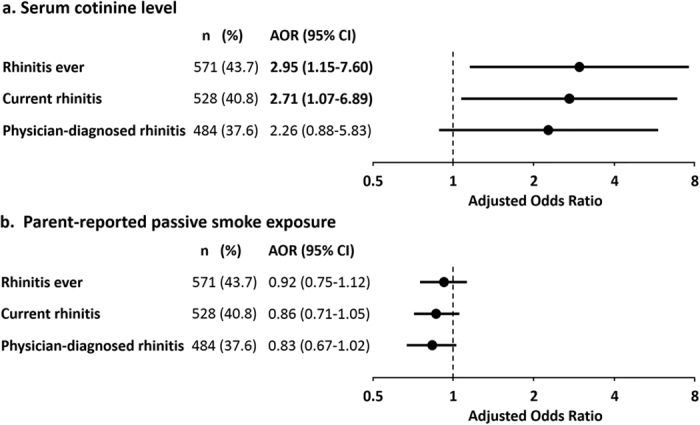
Plot of adjusted odds ratios with 95% CI (dot with bars) illustrating the association of serum cotinine levels (**a**) and parent-reported household passive smoke exposure (**b**) with rhinitis ever, current rhinitis, and physician-diagnosed rhinitis. *AOR*, adjusted odds ratio; *CI*, confidence interval.

The associations between tobacco smoke exposure and childhood rhinitis are illustrated in Fig. 1. After adjusting for confounders, significant positive associations were found between serum cotinine levels and rhinitis ever (adjusted odds ratio [AOR] = 2.95; 95% confidence interval [CI]: 1.15–7.60; *P* = 0.03) and current rhinitis (AOR = 2.71; 95% CI: 1.07–6.89; *P* = 0.04), while the association for physician-diagnosed rhinitis approaching borderline statistical significance (AOR = 2.26; 95% CI: 0.88–5.83; *P* = 0.09) (Fig. 1a). In contrast, we observed no significant association of exposure to household passive smoke which was determined from parental reports with rhinitis ever, current rhinitis, or physician-diagnosed rhinitis after adjusting for confounders (Fig. 1b).

We further performed interaction and stratified analyses using allergic sensitization, age, or gender, individually, to address whether these factors modified the association between serum cotinine levels and rhinitis. We found a borderline significant negative interaction between serum cotinine levels and allergic sensitization on current rhinitis (*P*_cotinine-allergic sensitization_ = 0.07). No significant interactions between serum cotinine levels and allergic sensitization were found on rhinitis ever and physician-diagnosed rhinitis, respectively. Similarly, we also found a borderline significant positive interaction between serum cotinine levels and age on current rhinitis (*P*_cotinine-age_ = 0.07), but not rhinitis ever or physician-diagnosed rhinitis. We found no statistically significant interaction between serum cotinine levels and gender on rhinitis ever, current rhinitis, or physician-diagnosed rhinitis. In addition, we categorized serum cotinine levels in tertiles and repeated analyses (Supplemental Table 1). Similar to the results presented in Fig. 1, significant association between current rhinitis and serum cotinine levels was found when treating serum cotinine levels in tertiles (AOR = 0.99; 95% CI: 0.75–1.31; *P* = 0.96 in the 2^nd^ tertile; AOR = 1.33; 95% CI: 1.01–1.77; *P* = 0.04 in the 3^rd^ tertile). Likewise, borderline significant association between physician-diagnosed rhinitis and serum cotinine levels was also found (AOR = 1.14; 95% CI: 0.86–1.52; *P* = 0.37 in the 2^nd^ tertile; AOR = 1.32; 95% CI: 0.99–1.76; *P* = 0.06 in the 3^rd^ tertile).

### Stratified analysis by allergic sensitization

We further stratified the analyses by allergic sensitization status (Table 2). Significant positive association of serum cotinine levels with increased risk of current rhinitis was observed among children without allergic sensitization (AOR = 6.76; 95% CI: 1.21–37.74; *P* = 0.03), but not among those with allergic sensitization (AOR = 1.18; 95% CI: 0.40–3.48; *P* = 0.76; Table 2). In addition, the results in Table 2 showed a borderline significant association for rhinitis ever among children without allergic sensitization (AOR = 4.64; 95% CI: 0.86–24.32; *P* = 0.07), but not in their counterpart (AOR = 1.53; 95% CI: 0.50–4.70; *P* = 0.45). We also categorized serum cotinine levels in tertiles and repeated analyses accordingly. The results are comparable to those treating cotinine as a continuous variable.

### Stratified analysis by age

We then performed stratified analyses to evaluate whether the detrimental effects of tobacco smoke exposure on rhinitis could be modified by age (Table 3). The results of age-stratified analyses indicated that serum cotinine levels were positively and significantly associated with increased risk of rhinitis ever (AOR = 3.34; 95% CI: 1.05–10.61; *P* = 0.04) and current rhinitis (AOR = 4.23; 95% CI: 1.28–13.97; *P* = 0.02), respectively, among teenagers aged equal to or more than 10 years, but no association was found in young children aged less than 10 years (Table 3). When active smokers were excluded in analyses, the association of serum cotinine levels with rhinitis ever (AOR = 5.01; 95% CI: 1.02–25.08; *P* = 0.05) and current rhinitis (AOR = 7.39; 95% CI: 1.46–37.41; *P* = 0.02) remained statistically significant among teenagers aged equal to or more than 10 years. Likewise, when classifying serum cotinine levels in tertiles and repeating analyses, the results are comparable to those treating cotinine as a continuous variable.

## Discussion

In this population-based sample of Asian children aged 5 to 18 years, the results suggested that exposure to tobacco smoke was significantly associated with rhinitis, particularly non-allergic rhinitis. Thus, the findings from this study provide epidemiological evidence that tobacco smoke exposure poses detrimental effects on respiratory health of Asian children in a population setting. More importantly, this study adds new evidence that the adverse effect of tobacco smoke exposure is mainly confined to non-allergic rhinitis and more pronounced in teenagers. This is one of the largest studies to date to assess the relationship of tobacco smoke exposure, as measured objectively by serum cotinine levels, with rhinitis in a community-based population of 1,315 children.

A global epidemiological study^3^ reveals that Taiwan has the highest prevalence of allergic rhinoconjunctivitis among children aged 6–7 years worldwide and the prevalence has markedly increased by 166% during a 7-year period. It has therefore drawn growing public attention to the need for effective strategies to reduce the healthcare burden of childhood allergies in this country. A recent study conducted by our group^12^ has demonstrated a dose-response relationship between tobacco smoke exposure and immunoglobulin E (IgE) sensitization to particular allergens including cockroaches, grass pollens, and certain foods among children in a general population. To date, a few studies have addressed the effects of tobacco smoke exposure on allergic rhinitis in children, though results have been inconsistent^13^,^14^,^15^,^16^,^17^,^18^,^19^,^20^. The reasons for this inconsistency may be partly explained by differences in extent of tobacco smoke exposure, populations, methods, and sample size. A majority of previous studies assessed exposure to tobacco smoke using questionnaires rather than using an objective assessment of cotinine levels. In a systematic review of 40 studies investigating the role of secondhand smoke in allergic rhinitis^20^, tobacco smoke exposure was evaluated using a cotinine/creatinine ratio in only 1 study^21^, whereas all other studies used a questionnaire to evaluate tobacco smoke exposure. Montano-Velazquez *et al*. found that tobacco smoke exposure was related to increased nasal resistance among adolescents with allergic rhinitis in Mexico^21^. In the current study, tobacco smoke exposure, as measured objectively by serum cotinine levels, was significantly related to rhinitis among school children in the community, which may have important public health implications. Both clinical and policy efforts to eliminate exposure to tobacco smoke might illuminate the potential to effectively and substantially decrease the health and economic burden of rhinitis in children.

Our findings are in line with previous studies. For example, in a study population of U.S. children and adolescents, Shargorodsky *et al*. indicated that tobacco smoke exposure was associated with increased prevalence of rhinitis symptoms in childhood^13^. In a study in Finland, parental smoking was associated with symptoms of perennial rhinitis in children^14^. Similarly, Zuraimi *et al*. found a relationship between home exposure to tobacco smoke and increased risks of current symptoms of rhinitis among preschool children in Singapore^15^. However, other studies have failed to find a link between tobacco smoke exposure and rhinitis in children^16^,^17^,^18^,^19^.

Of note, our results demonstrate that the association of tobacco smoke exposure with elevated risk of rhinitis is confined mainly to subjects without allergic sensitization. Some might hypothesize that allergic sensitization may act as a confounder rather than an effect modifier because parents of atopic children might smoke less. However, the fact that the prevalence of household passive smoke exposure and serum cotinine levels were both not significantly different between children with and without allergic sensitization suggests that confounding alone is unlikely to explain the observed association. Similar findings have been previously reported in Caucasian adults in which the association between active smoking and rhinitis was stronger in individuals without allergic sensitization^22^,^23^. A few plausible hypotheses can be raised. It is likely that tobacco smoke exposure may act as a pathogenic factor initiating the occurrence of rhinitis or exaggerating the symptoms of existing rhinitis. In contrast, tobacco smoke exposure could act as a modifying factor influencing the persistence of rhinitis. The current study, together with our previous work^12^ showing the relationship between tobacco smoke exposure and IgE sensitization clearly indicates that the harmful effect of tobacco smoke on rhinitis may be mediated through mechanisms other than IgE-mediated sensitization. The mechanisms behind the link between tobacco smoke exposure and non-allergic rhinitis are not yet well understood, but several mechanisms likely involved are described as follows. First, previous studies have documented the adverse effect of tobacco smoke exposure on nasal physiology and function by impairing mucociliary clearance^24^, reducing nasal volume^25^, and increasing nasal airway resistance^26^. Second, neurogenic inflammation resulting from tobacco smoke exposure may serve as a pathway distinct from antigen-driven, immune-mediated inflammation to produce vasodilatation, edema and infiltration of leukocytes^27^. Third, there is evidence demonstrating that tobacco smoke exposure can influence immune system signaling involving Toll-like receptors and the complement system which could favor the development of T helper 2 diseases^11^,^28^,^29^. Fourth, it remains possible that various genetic backgrounds, different environmental components and their interactions may have certain impact on the observed harmful effect of tobacco smoke exposure on rhinitis in the present study. Further investigation on this aspect would be merited.

Interestingly, the observed results from the current study are evident that adolescence, a life period characterized by various developmental changes, is a period of heightened vulnerability to the harmful effects of tobacco smoke exposure on nasal health. Previous studies reported that average age for children entering puberty was approximately 10 years^30^,^31^. Therefore, we have classified children aged over and under 10 years in this study. Although the underlying mechanisms remain unclear, this phenomenon could be linked to puberty-related factors such as hormones, or perhaps other environmental factors and their gene-environment interactions^32^. Nevertheless, further longitudinal studies are needed to examine whether adolescence may influence the relationship between tobacco smoke exposure and respiratory diseases in a more harmful way than their early life.

Notably, we have noted the discrepancy between results obtained from cotinine measurements and those analyzed from parental reports of household passive smoke exposure. It is likely that household tobacco smoke exposure assessment based on parental reports may underestimate the true extent of exposure and therefore may fail to discern the detrimental effects of children’s exposure to tobacco smoke. In contrast to parental reports of household passive smoke exposure, cotinine measurements in body fluids is currently considered as the gold standard of tobacco smoke exposure assessment. From the literature, parental reports of household passive smoke exposure have been found to be potentially biased for some reasons, including under-reporting of household smoking status and underestimation of exposure outside the household^33^, which taken together could have contributed to an underestimation of the extent of passive tobacco smoke exposure and could have led to the observed discrepancy in this study.

The present study has several strengths. First, the study was performed in a representative cohort of children recruited from the general population with a large sample size, a wide age distribution, and a high participation rate. Second, the quantitative measurement of serum cotinine levels as an objective indicator of tobacco smoke exposure is another strength of the study, specifically avoiding the risk of misclassification or underestimation of tobacco smoke exposure. Of note, several studies including ours^12^,^34^ have documented even though cotinine can be served as the best available biomarker reflecting recent exposure to tobacco smoke, cotinine could only be considered as a proxy for long-term (or cumulative) tobacco smoke exposure. Third, the objective measure of allergic sensitization allows for a better phenotypic characterization of rhinitis. However, some potential limitations should be also noted. First, it remains to be confirmed whether the results in this population of Asian children in Taiwan are generalizable to other ethnic populations or other geographic regions. Second, the cross-sectional nature of this study is a limitation which precludes our ability to make causal inferences about the observed associations. Further long-term cohort studies to examine longitudinal/ temporal impact of tobacco smoke exposure on rhinitis in children would be merited. Third, as indicated above, tobacco smoke exposure has impact on Toll-like receptors and the complement system which could favor the development of T helper 2 diseases^11^,^28^,^29^. However, we did not measure expression levels of Th1 and Th2 cytokines in the present study. It will be merited to further investigate the relationship between serum cotinine levels and Th1 and Th2 cytokines. Fourth, we did not provide an optimal cut-off threshold of serum cotinine to differentiate between active and passive smokers in our study population. Further investigation would be needed to determine cotinine thresholds for discriminating active and passive adolescent smokers.

In conclusion, this population-based study demonstrates new evidence for the significant association of exposure to tobacco smoke with increased risk of rhinitis in children in the general population, while the detrimental effect is mainly confined to non-allergic rhinitis and more pronounced in adolescents than in young children. Thus, the findings from this study provide supporting evidence for the adverse effects of tobacco smoke exposure on the respiratory health of Asian children in a population setting. General public awareness of the harmful effects of tobacco smoke exposure together with operational clinical and public health strategies will be of importance to effectively eliminate exposure to tobacco smoke and hopefully might contribute to decreasing the health and economic burden of childhood rhinitis, particularly in countries where rhinitis is epidemic and tobacco smoke exposure is common.

## Methods

### Study participants

**Figure 2:**
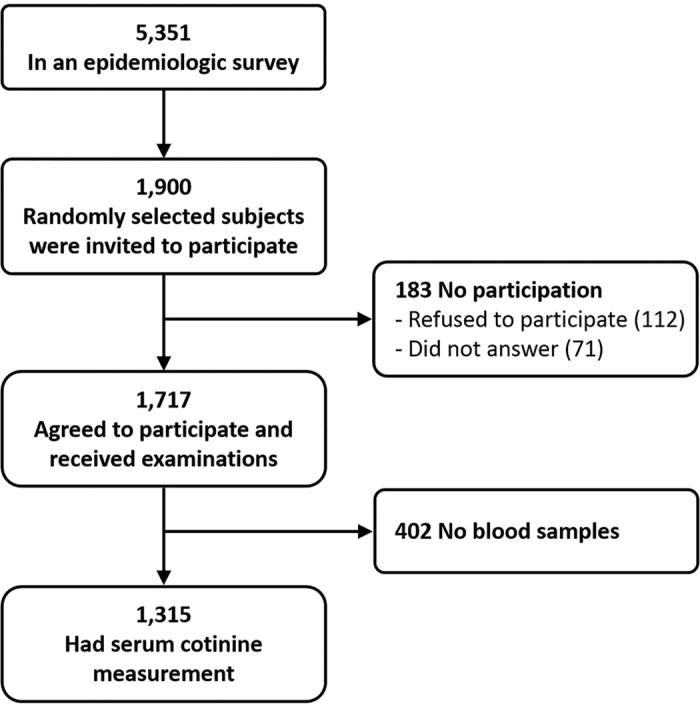
Schematic presentation of the recruitment process of the study participants.

A total of 1,315 children recruited from the PATCH study, part of a prospective population-based cohort study, were included in the present study. The PATCH study launched in 2007, aiming to investigate the epidemiology and predictive factors related to asthma and allergies in children. Detailed descriptions of the study population, data collection and previous findings in publications derived from the PATCH study have been reported elsewhere^4^,^9^,^12^,^35^,^36^,^37^,^38^,^39^. The flow chart for subject recruitment is depicted in Fig. 2. Briefly, a school-based sample of 5,351 children initially participated in an International Study of Asthma and Allergies in Childhood (ISAAC) epidemiologic survey^4^. Among those, a random sample of 1,900 participants were selected and invited to participate in a thorough examination and 1,717 agreed to participate, representing a participation rate of 90.4%. Parents of all participants answered a questionnaire regarding demographic data, household passive smoke exposure, and general health information. We further measured serum levels of cotinine and allergen-specific IgE in 1,315 study children whose parents agreed to provide their child’s blood sample. No statistically significant differences between the characteristics of the study subjects and the original cohort members were found, indicating this sampling cohort is representative of the general population. This study was approved by the Institutional Review Board of Chang Gung Medical Foundation (No. 102-5592A3) and the parents of all participants provided written informed consents. All experiments in this study were performed in accordance with the relevant guidelines and regulations.

### Exposure assessment

We determined serum levels of cotinine, a metabolite of nicotine, among 1,315 study children using enzyme-linked immunosorbent assay (ELISA), according to the manufacturer’s instructions (Calbiotech, Spring Valley, California). Exposure to household passive smoke was also assessed based on the questionnaire data collected by parental reports.

### Outcome assessment

Previous and current clinical symptoms of rhinitis and physician diagnosis of rhinitis were obtained using a modified ISAAC questionnaire reported by parents of the study participants^4^,^40^. Allergic sensitization was defined as a positive Phadiatop Infant test result (≥0.35 PAU/l). Phadiatop^®^ Infant (Phadia, Uppsala, Sweden) is a reliable alternative to skin prick tests for detecting allergen-specific IgE against the following allergens: house dust mite, cat, dog, birch, timothy, ragweed, wall pellitory, egg white, cow’s milk, peanut, and shrimp^41^.

### Statistical analysis

Descriptive statistics were reported either as counts and the corresponding percentages, or by mean and the corresponding standard deviation (SD), respectively. Kruskal-Wallis equality-of-populations rank test was used to test the distribution of serum cotinine levels between boys and girls. Logistic regression analyses were applied to determine the association between tobacco smoke exposure (either serum cotinine levels or household passive smoke [yes/no]) and rhinitis, with and without adjustment of covariates such as age, gender, and body mass index (BMI). To evaluate potential effect modification, we performed subgroup analysis, stratified by allergic sensitization, age and gender, separately. To obtain approximate normality, we took log_10_-transformation of serum cotinine values for all analyses. A *P*-value less than 0.05 was declared to be statistically significant. All analyses were carried out using STATA 11.0 software (StataCorp, College Station, TX, USA).

## Additional Information

**How to cite this article**: Yao, T.-C. *et al*. Exposure to tobacco smoke and childhood rhinitis: a population-based study. *Sci. Rep.* **7**, 42836; doi: 10.1038/srep42836 (2017).

**Publisher's note:** Springer Nature remains neutral with regard to jurisdictional claims in published maps and institutional affiliations.

## Supplementary Material

Supplemental Information

## Figures and Tables

**Table 1:** Demographic and clinical characteristics of the 1,315 study participants.

Characteristic	All (*N* = 1,315)
Age, years (mean ± SD)	10.3 ± 2.7
Gender, *n* (male/female) (%)	645/670 (49.0)
Anthropometric measurement	
Height, cm (mean ± SD)	138.9 ± 14.8
Weight, kg (mean ± SD)	37.2 ± 13.4
Body mass index, kg/m^2^ (mean ± SD)	18.7 ± 3.6
Household passive smoking, *n* (yes/no) (%)	692/586 (54.1)
Serum cotinine level, ng/ml (mean ± SD)	1.51 ± 2.99
Rhinitis ever, *n* (yes/no) (%)	571/735 (43.7)
Current rhinitis, *n* (yes/no) (%)	528/767 (40.8)
Physician-diagnosed rhinitis, *n* (yes/no) (%)	484/803 (37.6)
Allergic sensitization, *n* (yes/no) (%)	749/562 (57.1)

**Table 2:** Association of serum cotinine levels with rhinitis in 1,315 study participants, stratified by allergic sensitization*.

	No. (%)	Crude OR (95% CI)	*P*	Adjusted OR (95% CI)†	*P*†
Children without allergic sensitization (*N* = 562)					
Rhinitis ever	143 (25.6)	4.19 (0.85–20.55)	0.08	4.64 (0.86–24.32)	0.07
Current rhinitis	122 (22.0)	**6.23 (1.17–33.20)**	**0.03**	**6.76 (1.21–37.74)**	**0.03**
Physician-diagnosed rhinitis	126 (22.8)	1.69 (0.34–8.37)	0.52	1.63 (0.31–8.61)	0.56
Children with allergic sensitization (*N* = 749)					
Rhinitis ever	426 (57.3)	1.20 (0.42–3.42)	0.73	1.53 (0.50–4.70)	0.45
Current rhinitis	403 (54.7)	1.01 (0.36–2.84)	0.99	1.18 (0.40–3.48)	0.76
Physician-diagnosed rhinitis	356 (48.8)	1.59 (0.50–5.03)	0.43	1.81 (0.55–6.00)	0.33

*OR*, odds ratio; *CI*, confidence interval.

^*^Serum cotinine levels were log_10_-transformed and treated as a continuous variable. *P*-values less than 0.05 are in bold. Four subjects had missing data on allergic sensitization.

^†^Adjusted covariates included age, gender, and body mass index.

**Table 3:** Association of serum cotinine levels with rhinitis in 1,315 study participants, stratified by age*.

	No. (%)	Crude OR (95% CI)	*P*	Adjusted OR (95% CI)†	*P*†
Children aged ≥10 years (*N* = 682)					
Rhinitis ever	297 (44.0)	**3.37 (1.09–10.42)**	**0.04**	**3.34 (1.05–10.61)**	**0.04**
Current rhinitis	278 (41.4)		**0.02**	**4.23 (1.28–13.97)**	**0.02**
Physician-diagnosed rhinitis	245 (36.7)	2.74 (0.90–8.36)	0.08	2.62 (0.83–8.22)	0.10
Children aged <10 years (*N* = 633)					
Rhinitis ever	274 (43.4)	1.25 (0.23–6.80)	0.79	2.35 (0.41–13.36)	0.33
Current rhinitis	250 (40.1)	0.55 (0.09–3.41)	0.53	0.88 (0.14–5.59)	0.89
Physician-diagnosed rhinitis	239 (38.6)	0.97 (0.16–5.88)	0.97	1.74 (0.27–11.07)	0.56

*OR*, odds ratio; *CI*, confidence interval.

^*^Serum cotinine levels were log_10_-transformed and treated as a continuous variable. *P*-values less than 0.05 are in bold.

^†^Adjusted covariates include gender and body mass index.
