# Supplementary material for: Exposure to tobacco smoke and childhood rhinitis: a population-based study
Source: Sci Rep. 2017 Feb 16;7:42836. doi: 10.1038/srep42836 (PMC5311963; doi:10.1038/srep42836)
Supplement: Supplemental Information [file srep42836-s1.pdf]

Supplementary Information

Exposure to tobacco smoke and childhood rhinitis: a population-based study

Tsung-Chieh Yao, MD, PhD[1,2,3]; Su-Wei Chang, PhD[4]; Wei-Chiao Chang, PhD[5]; Ming-Han Tsai, MD, PhD[3,6]; Sui-Ling Liao, MD[3,6]; Man-Chin Hua, MD[3,6]; Shen-Hao Lai, MD[3,7]; Kuo-Wei Yeh, MD[1,3]; Yu-Lun Tseng, BS[1,3]; Wan-Chen Lin, MS[1,3]; Hui-Ju Tsai, MPH, PhD[8,9,10]; Jing-Long Huang, MD[1,3].

[1]Division of Allergy, Asthma, and Rheumatology, Department of Pediatrics, Chang Gung Memorial Hospital and Chang Gung University College of Medicine, Taoyuan, Taiwan
[2]Chang Gung Immunology Consortium, Chang Gung Memorial Hospital and Chang Gung University College of Medicine, Taoyuan, Taiwan
[3]Community Medicine Research Center, Chang Gung Memorial Hospital at Keelung, Keelung, Taiwan
[4]Clinical Informatics and Medical Statistics Research Center, Chang Gung University College of Medicine, Taoyuan, Taiwan
[5]Department of Clinical Pharmacy, College of Pharmacy, Taipei Medical University, Taipei, Taiwan
[6]Department of Pediatrics, Chang Gung Memorial Hospital at Keelung, Keelung, Taiwan
[7]Division of Pediatric Pulmonology, Department of Pediatrics, Chang Gung Memorial Hospital, Taoyuan, Taiwan
[8]Division of Biostatistics and Bioinformatics, Institutes of Population Health Sciences, National Health Research Institutes, Miaoli, Taiwan
[9]Department of Pediatrics, Feinberg School of Medicine, Northwestern University, Chicago, IL, USA
[10]Department of Public Health, China Medical University, Taichung, Taiwan

Supplemental Table 1. Association of serum cotinine levels (in tertiles) with rhinitis in 1,315 study participants*.

	No. (%)	Crude OR (95% CI)	*P*	Adjusted OR (95% CI)†	*P*†
**Rhinitis ever**	566 (43.6)				
**1st tertile**	189 (33.4)	Ref		Ref	
**2nd tertile**	172 (30.4)	1.03 (0.78-1.35)	0.84	1.02 (0.78-1.34)	0.90
**3rd tertile**	205 (36.2)	1.18 (0.91-1.54)	0.21	1.26 (0.95-1.67)	0.10
**Current rhinitis**	524 (40.8)				
**1st tertile**	175 (33.4)	Ref		Ref	
**2nd tertile**	157 (30.0)	1.00 (0.76-1.32)	0.99	0.99 (0.75-1.31)	0.96
**3rd tertile**	192 (36.6)	1.23 (0.94-1.60)	0.13	**1.33 (1.01-1.77)**	**0.04**
**Physician-diagnosed rhinitis**	481 (37.6)				
**1st tertile**	154 (32.0)	Ref		Ref	
**2nd tertile**	150 (31.2)	1.14 (0.86-1.51)	0.35	1.14 (0.86-1.52)	0.37
**3rd tertile**	177 (36.8)	1.29 (0.98-1.70)	0.07	1.32 (0.99-1.76)	0.06

OR, odds ratio; *CI*, confidence interval.

*Serum cotinine levels were treated as a categorical variable (in tertiles). *P*-values less than 0.05 are in bold.

†Adjusted covariates included age, gender, and body mass index.
